# Switching diets after 6-months does not result in renewed weight loss: a secondary analysis of a 12-month crossover randomized trial

**DOI:** 10.1038/s41598-024-60547-z

**Published:** 2024-04-29

**Authors:** Matthew J. Landry, Catherine P. Ward, Kristen M. Cunanan, Priya Fielding-Singh, Anthony Crimarco, Christopher D. Gardner

**Affiliations:** 1 Stanford Prevention Research Center, School of Medicine, Stanford University, Stanford, CA USA; 2 Department of Population Health and Disease Prevention, Program in Public Health, University of California, Irvine, 856 Health Sciences Rd., Irvine, CA 92697 USA; 3 Quantitative Science Unit, School of Medicine, Stanford University, Stanford, CA USA; 4 Sandberg Goldberg Bernthal Family Foundation, San Francisco, CA USA; 5 Division of Gastroenterology and Hepatology, Stanford Center for Clinical Research, Stanford University, Redwood City, CA USA

**Keywords:** Obesity, Clinical trials

## Abstract

Weight change trajectory from diet and lifestyle interventions typically involves rapid weight loss followed by a weight plateau after approximately 6 months. Changing from one weight-loss diet to another at the time of the plateau could instigate renewed weight loss. Therefore, our secondary analysis aimed to assess trajectory of weight loss in a 12-month, randomized, cross-over study. Forty-two adults were randomized to eat a healthy low-fat or healthy low-carbohydrate diet for 6 months then switched to the opposite diet for an additional 6 months. Regardless of diet assignment, participants experienced rapid initial weight loss, which slowed between 3 to 6 months. After switching diets at 6 months, weight modestly decreased until 9 months, but at a rate slower than the initial 3 months and slower than the rate from 3 to 6 months. This suggests that the weight loss plateau typically seen at 6 months is physiological and cannot be overcome by simply switching to a different weight-loss diet.

## Introduction

Preventing and treating obesity has remained a difficult public health challenge to address. Typically, only 20 percent of individuals with overweight or obesity maintain their initial weight loss after 5 years^1,2^. The complexities of long-term weight management are multifactorial and include the interaction between our obesogenic environment, physiology, and health behaviors^1^. The usual trajectory for weight change from diet and lifestyle interventions involves a period of rapid weight loss in the early stages of the intervention followed after a few months by a weight plateau (static phase of weight loss), and weight regain within a year or two^3,4^. Many diet interventions specifically report weight loss plateaus after approximately 6 months^4–10^. Numerous physiological explanations have been given to account for the weight-loss plateau, including both orexigenic and anorexigenic adaptions that influence appetite, satiety, and energy homeostasis^11–13^.

It is important for researchers and clinicians to understand the extent to which weight-loss plateaus are physiological versus psychological in order for them to help individuals maintain the weight they have lost or continue to lose weight. For example, outdated guidance for sustaining weight loss through moderate caloric reductions (i.e., the so called 3500 kcal per pound rule) continues to be prescribed to participants, even though this advice neglects the body’s decrease in energy expenditure from initial weight loss and increase in appetite^14–16^. Another explanation for participant weight loss plateaus, which is more psychological than physiological, is that of “diet fatigue,” or boredom with one’s diet^17–19^. The premise of diet fatigue is that people begin to lose interest in dieting from routinely eating the same specific foods^20^. Therefore, people lessen an adherence to their diet (dietary disinhibition) and stop maintaining their good habits, including dietary self-monitoring. Consistent dietary self-monitoring is predictive of weight loss^21–23^, but few people consistently commit to monitoring their diet long term^24^. After becoming unmotivated to consistently monitor their diets, dieters can regain their lost weight^25^. Changing weight-loss diets (e.g., from a low-fat diet to a low-carbohydrate diet) could help prevent diet fatigue because it introduces new foods into a person’s everyday diet, which may increase motivation for dietary self-monitoring. However, while diet fatigue has been discussed anecdotally, we are not aware of any dietary interventions that have assessed this theory.

Therefore, in this secondary analysis, our objective was to characterize and evaluate participant’s percent change in weight before and after switching from a healthy low-carbohydrate (LC) diet to a healthy low-fat (LF) diet (or vice versa) among overweight and obese adults using data from a parent 12-month randomized, crossover trial. The parent study was originally designed to assess 6-month differential weight loss responses to a LF vs. LC diet by insulin resistance status among overweight and obese individuals^26^. It served as a pilot study for The Diet Intervention Examining the Factors Interacting with Treatment Success (DIETFITS) trial by developing the “Limbo-Titrate-Quality” approach to defining a healthy LF and LC diet^26^. An optional crossover was incorporated into the study design to increase the efficiency of recruitment. In this study, 42 of the original 61 participants switched diets midway through the study—half from LF to LC, and half from LC to LF—making it possible to examine whether that switch instigated renewed weight loss.

## Results

### Sample characteristics

**Figure 1:**
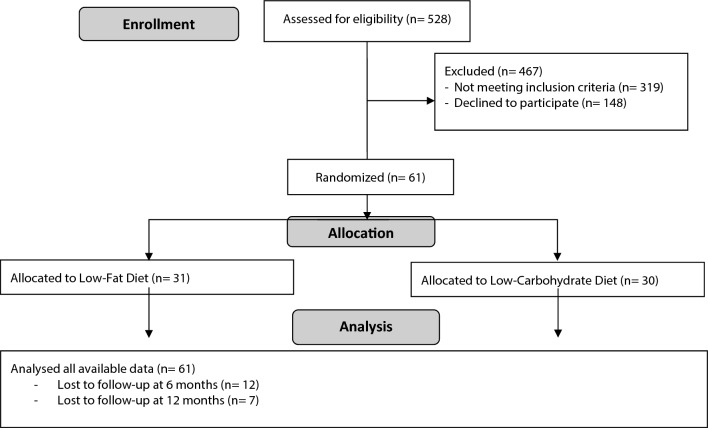
Consort diagram of participant flow. As prespecified in the statistical analysis plan, the primary analysis includes all available data.

Of the 61 participants enrolled, 49 (80%) completed the 6-month protocol and 42 (69%) completed the full 12-month protocol (which included the cross-over). A consort diagram of participant flow is provided in Fig. 1. Baseline characteristics of participants overall and stratified by diet order are presented in Table 1. Briefly, participants on average were 42 years of age, 64% female, 87% White, and weighed 97 kg. Baseline characteristics of participants stratified by insulin resistance (IR) and insulin sensitivity (IS) are presented elsewhere^26^.

**Table 1:** Baseline participant characteristics.

Variable^a^	Total	Low-Fat → Low-Carbohydrate	Low-Carbohydrate → Low-Fat
	N = 61	N = 31	N = 30
Women [n (%)]	37 (63.8)	18 (64.3)	19 (63.3)
Age (years)	42.3 (6.0)	42.1 (5.6)	42.6 (6.5)
Education (years)	16.1 (2.4)	16.0 (2.80)	16.3 (1.9)
Race [n (%)]			
White	53 (86.9)	25 (80.6)	28 (93.3)
Black or African American	3 (4.9)	3 (9.7)	0 (0.0)
Asian	5 (8.2)	3 (9.7)	2 (6.7)
Ethnicity (Hispanic) [n (%)]	10 (16.4)	3 (9.7)	7 (23.3)
Weight (kg)	97.4 (16.3)	100.1 (16.2)	94.5 (16.2)
LDL-C (mg/dl)	112.3 (26.1)	114.3 (25.5)	110.3 (27.0)
HDL-C (mg/dl)	47.0 (13.8)	47.2 (14.3)	46.7 (13.4)
Triglycerides (mg/dl)	138.8 (71.9)	131.3 (59.6)	146.6 (83.1)
Fasting Glucose (mg/dl)	102.7 (13.0)	104.7 (15.2)	100.8 (10.1)
Insulin (µu/ml)	19.5 (8.9)	17.8 (6.1)	21.6 (11.3)

*LDL-C* low-density lipoprotein, *HDL-C* high-density lipoprotein.

^a^Data are expressed as mean (standard deviation), unless otherwise indicated.

### Dietary adherence

**Figure 2:**
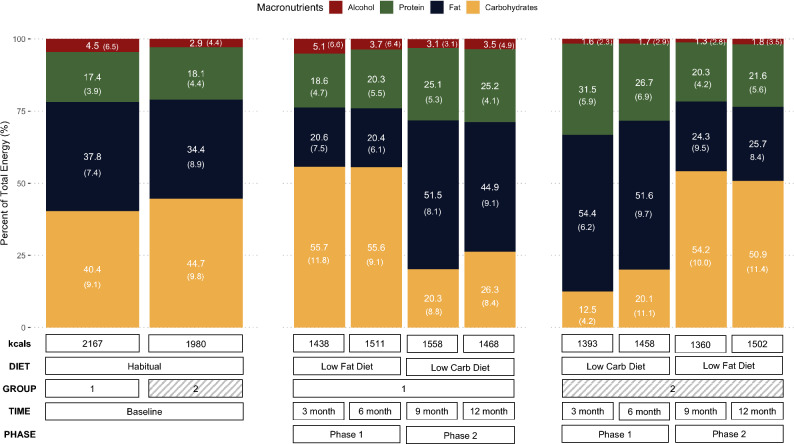
Dietary assessment arranged by group. Proportions of alcohol, protein, fat, and carbohydrates at baseline, 3-, 6-, 9-, and 12-months.

Participants in both diet arms made and sustained substantial dietary changes as assessed at 3, 6, 9, and 12 months, relative to baseline (Fig. 2). Participants changed the macronutrient composition of their diet at 6 months as they switched from a LF to LC diet (and vice versa). Data suggest that participants similarly adhered to dietary recommendations for both diets regardless of diet order. Irrespective of diet assignment, reported caloric intake was decreased from baseline levels at 3, 6, 9, and 12 months. Also notable was the protein intake was > 25% of total energy in both groups when consuming a LC diet.

### Change in weight

**Figure 3:**
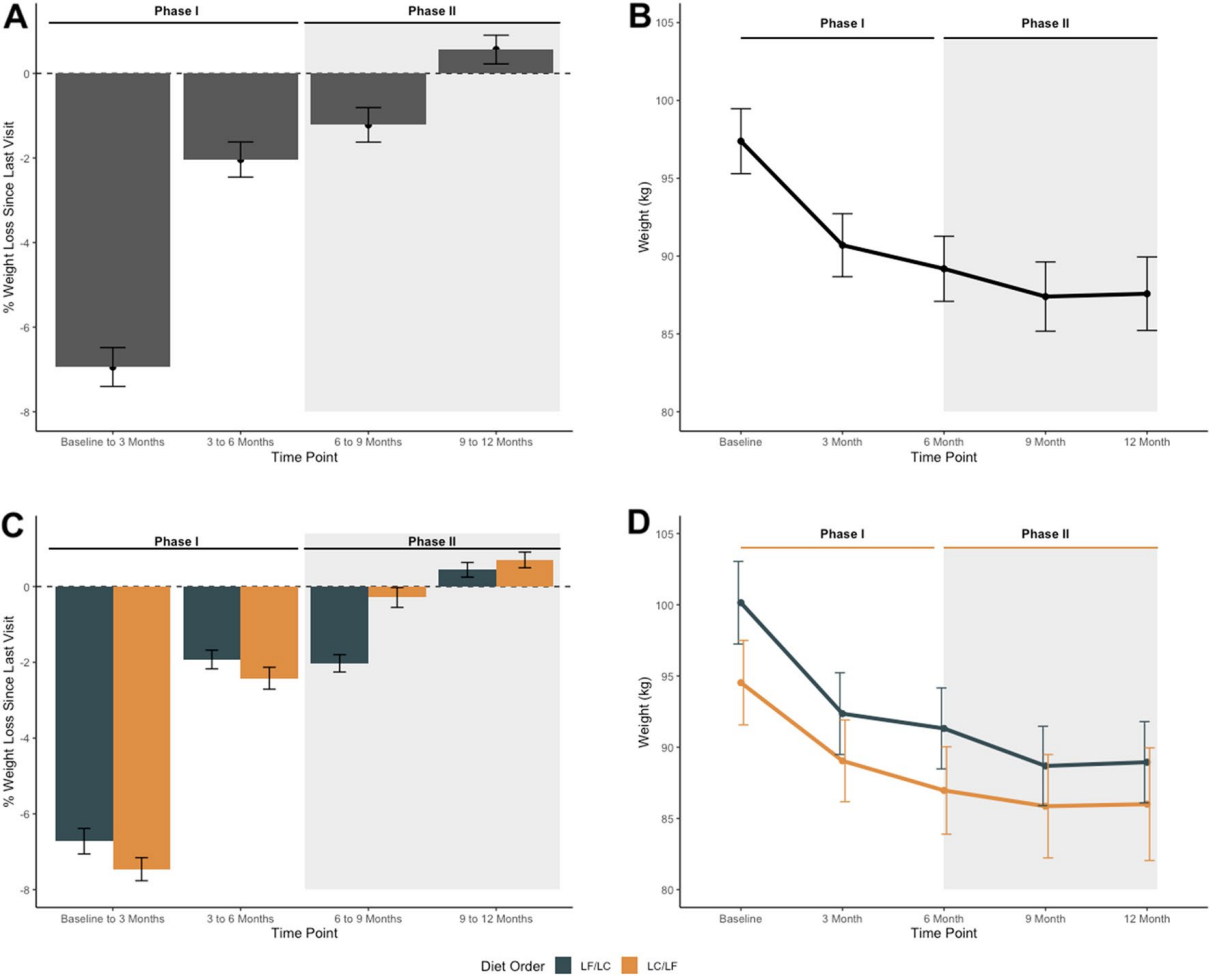
Average change in weight. (**A**) Percent weight loss since last visit and (**B**) absolute weight (kg). Sample size at each timepoint: Baseline (n = 61), 3-months (n = 52), 6-months (n = 49), 9-months (n = 44), and 12-months (n = 42). *LF* Low-Fat, *LC* Low-Carbohydrate.

The average percent weight change (since previous visit) and 95% CI for each time point is presented in Fig. 3A; similarly, the absolute mean weight is presented in Fig. 3B. In both Fig. 3A,B, we observed, on average, that participant’s weight loss was rapid between baseline and 3 months, and then slowed between 3 and 6 months. On average, in the first 3-months participants percent weight change was − 7% (95% CI − 8%, − 6%), and between 3 and 6 months percent weight change slowed to − 2% (95% CI − 3%, − 1%) (Fig. 3A). Following the crossover, on average, participants displayed a − 1% (95% CI − 2%, − 0.4%) percent weight change between 6 and 9 months. Between 9 and 12 months the percent weight change was negligible, a modest increase of 0.6% (95% CI − 0.1%, 1.3%). One notable subgroup is that between 6 and 9 months, participants who switched from LF to LC maintained an average percent weight change of − 2% while participants who switched from LC to LF experienced a nearly 0% average percent weight change (Fig. 3C,D).

Based on the primary linear mixed model, on average, after switching their diet, participants experienced a relative 0.9% (95% CI − 0.1, 1.9) increase in weight change over 3 months, compared with the 3 months prior to the change in diet (Supplementary Table 1). In other words, rather than accelerating or restarting weight loss in the second phase, the change in diet was accompanied by a relative decrease in the rate of weight loss. When stratified by diet order, on average, participants in the LF diet first arm did not experience a plateau or loss but rather a similar change in percent weight change from 6 to 9 months relative to 3–6 months (− 0.1%, 95% CI − 1.5%, 1.3%); whereas participants with the LC diet first essentially nullified their 3 to 6 month percent weight loss during the 6- to 9-month phase after switching diets (a relative change of 2.2%, 95% CI 0.7%, 3.6%) (Supplementary Table 2). This is also displayed in Fig. 3C,D. Linear mixed model estimates of absolute weight loss are provided in Supplementary Table 3. On average, weight decreased by 7 kg (− 8.4, − 5.6) for the first 3 months; and decreased by 8.9 kg for the next 3 months (relative to baseline). After the diet change, on average, weight decreased by 10.2 kg (− 11.6, − 8.7) for the first 3 months of the second phase (relative to baseline); and decreased by 9.9 kg (− 11.4, − 8.4) for the next 3 months (relative to baseline). That is, on average, participants’ observed a 1.3 kg decrease in weight after switching diets.

Participants’ individual trajectory of absolute weight and percent weight change are shown in Supplementary Fig. 1 and stratified by diet order in Supplementary Fig. 2 and by diet order and insulin resistance in Supplementary Fig. 3. These figures display some possible differential trends, but also highlight the small sample size and large variability when considering these subgroups.

### Changes cardiovascular clinical measures

**Figure 4:**
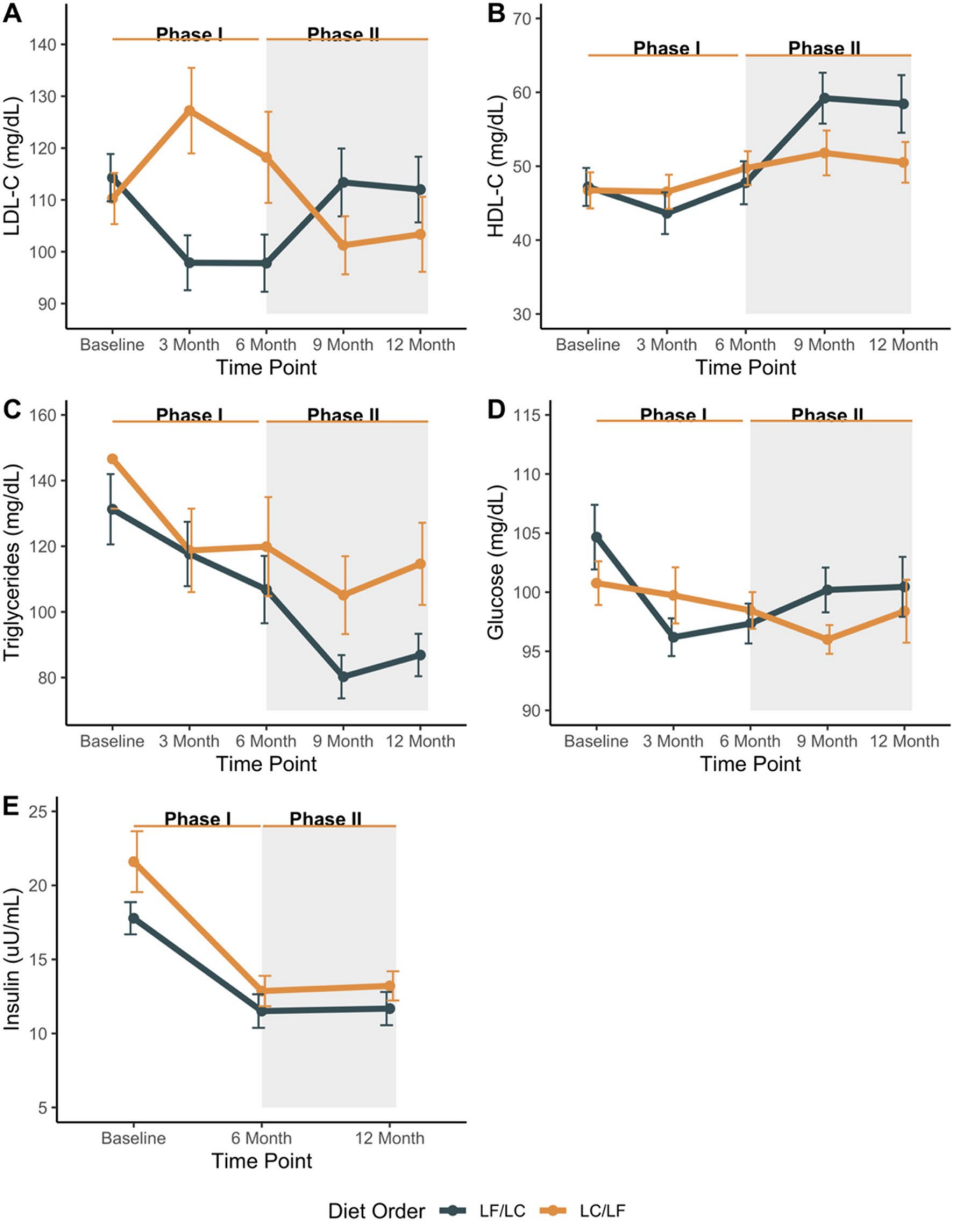
Average change in cardiovascular clinical measurements at each time point by diet order. (**A**) LDL-C, (**B**) HDL-C, (**C**) Triglycerides, (**D**) Glucose, and (**E**) Insulin.

Changes in cardiovascular clinical measures are shown in Fig. 4 and provide an indirect measurement of adherence to study diets. Average trends are described below and statistical differences between measures by diet group are presented elsewhere^26^. As anticipated, LDL-C was elevated at 3 months relative to baseline for participants in the LC/LF diet order and decreased when participants switched to the LF diet at 6 months (Fig. 4A). The opposite was observed for participants in the LF/LC diet order for which LDL-C dropped at 3 months and increased between 6 and 9 months when participants began the LC diet. For both diet orders, HDL-C remained similar between baseline and 6 months, although in absolute values the levels decreased modestly for the group assigned LF diet first (Fig. 4B). After the diet crossover, HDL-C increased for participants in the LF/LC diet order when beginning a LC diet, and the patterns display another relative crossover. Triglycerides decreased for both diet orders between baseline and 9 months, which was likely largely attributable to weight loss (Fig. 4C). In the second phase, the decrease in triglycerides was significantly different and more substantial for the group eating the LC diet second, despite relative weight stability in the second phase. Participants in the LC/LF diet order observed a gradual decrease in glucose between baseline and 9 months with a small increase between 9 and 12 months (Fig. 4D). Participants in the LF/LC diet order saw a rapid decrease in glucose between baseline and 3 months and a gradual increase between 3 and 12 months. Insulin levels decreased for both diet orders between baseline and 6-months; however, insulin levels plateaued in both groups after the 6-month crossover (Fig. 4E). Mean values for cardiovascular clinical measures at each time point stratified by diet order are presented in Supplementary Table 4 and linear mixed model estimates for cardiovascular clinical measures are presented in Supplementary Table 5.

### Weight stable or gaining

A participant was considered weight stable or gaining if their percent weight change at the end of phase 1 (6 month timepoint) was greater than or equal to − 2 percent (weight loss) relative to the weight change at the 3-month timepoint. Subsequently, 27 (44%) of study participants were considered weight stable or gaining. Among this cohort, on average, participants that were weight stable or gaining experienced a decrease, − 1.0% (95% CI − 2.3, 0.2), in percent weight change over 3 months, relative to the percent weight change during the 3 months prior to the change in diet (Supplementary Table 6).

### DIETFITS as a historical control

**Figure 5:**
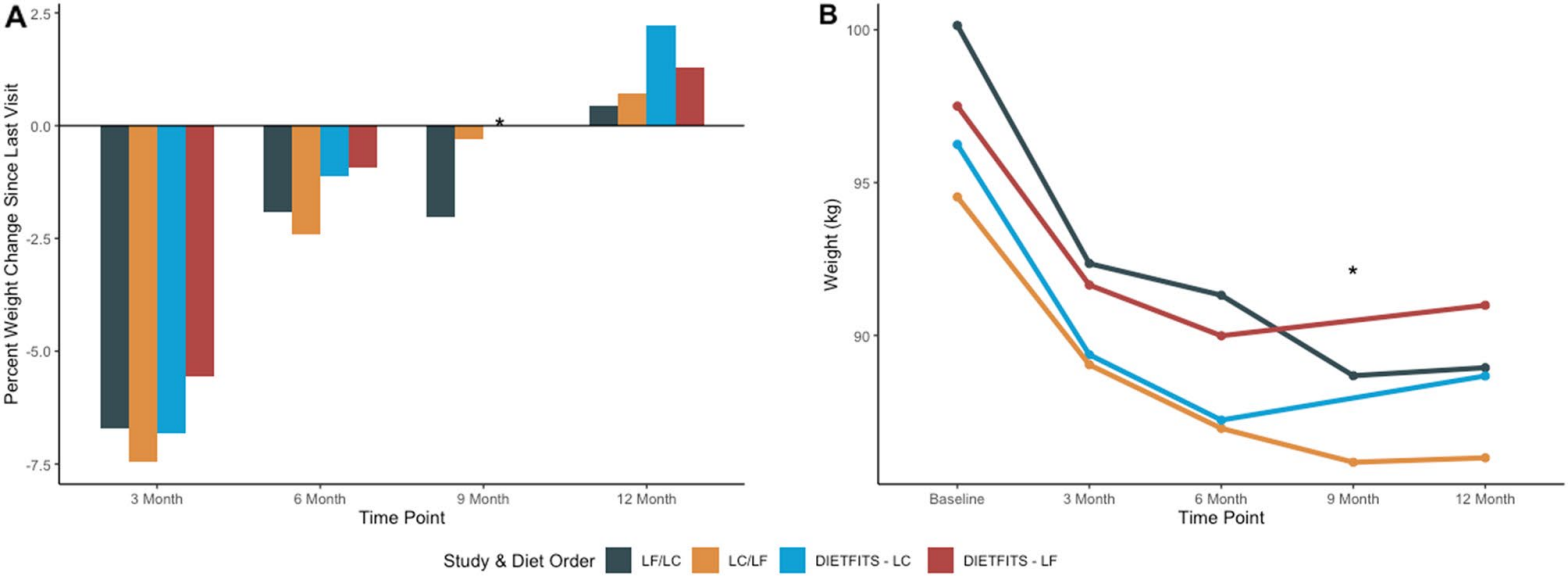
Average percent weight change in a 12-month crossover trial comparing low-fat to low-carbohydrate (with crossover at 6 months) overlaid with the DIETFITS 12-month parallel arm dietary intervention comparing low-fat to low-carbohydrate. Weight data were not available at 9 months for DIETFITS trial. Sample size at 12-months LF/LC (n = 20); LC/LF (n = 22); DIETFITS-LC (n = 304); DIETFITS-LF (n = 305). *LF* Low-Fat, *LC* Low-Carbohydrate, *DIETFITS* The Diet Intervention Examining the Factors Interacting with Treatment Success.

In our crossover study, participants on LF and LC experienced a sharp decrease in weight during the first 3 months of their first diet. The rate of weight loss slowed between 3 and 6 months but did not plateau, on average. Based on the weight trajectories, one might expect that participants may have continued to lose weight beyond 6 months had they not switched to a different diet. However, data from DIETFITS^10^ a 12-month randomized controlled trial involved a dietary intervention that was essentially identical (our current trial was a pilot trial for DIETFITS), which was used as a historical control (Fig. 5). In DIETFITS, participants had a similar trajectory of weight loss between 0 and 6 months, rapid between 0 and 3 months, then slowly decreasing between 3 and 6 months. However, unlike the present crossover study, in the DIETFITS trial, participants remained on either a LF or LC diet for 12 months, twice the duration of this study. Between 6 and 12 months, DIETFITS participants were able to maintain most of their weight loss on average.

## Discussion

This study was a secondary analysis that assessed changes in body weight among overweight or obese adults that were randomly assigned to either a healthy LF or healthy LC diet and then switched diets after 6 months. Although not designed to address the hypothesis being tested here, the availability of data allowed for an exploration of a novel hypothesis regarding the typical weight loss plateau in weight-loss diet studies at 6 months^4,27^. By having participants change their diet after 6 months, we hypothesized that there was potential to reinvigorate weight loss by restarting interest in diet monitoring and increasing attention on intentional diet choices^17–19,21–23^. However, switching from one healthy weight loss diet to another, did not accelerate or instigate renewed weight loss beyond the 6-month time point.

Over the last several decades, research within long-term diet interventions, whether targeting macronutrients, calories, or food patterns, are capable, at least to a degree, of achieving similar short-term success in weight loss^28^. However, as people progressively lose more weight, they fight an increasing battle against the physiological and psychological responses that oppose weight loss^1^. More recent findings show that the level of dietary adherence and the overall calorie deficit, rather than the composition of the diet, is be a better predictor of magnitude of initial weight loss^27^. However, adherence to dietary modifications is a well-known challenge within dietary interventions^29^. A participant’s adherence to a diet typically begins to wane after around 6 months^4,30,31^. There are many reasons for this, including the perceived costs of adherence gradually exceeding the perceived benefits, suggesting that diet fatigue can occur, allowing a return of negative behaviors and habits that predate weight loss after a period of successful control^32,33^.

Dieters’ frustration from weight loss plateau is well established^1^. Prior research indicates that after a period of rapid weight loss the body responds by decreasing energy expenditure, fat oxidation, and anorexigenic hormones like leptin^11^. As an individual loses weight, their resting metabolic rate also decreases because there is less mass to support^13^. At the same time, the body also responds to weight loss by increasing appetite-stimulating hormones like ghrelin, gastric inhibitory polypeptide, and pancreatic polypeptide^12^. In the absence of ongoing efforts to restrain food intake following weight loss, there is a proportional increase in appetite. This typically results in dieters eating about 100 cal per day above baseline levels per kilogram of lost weight, thus accelerating their weight regain^16^. Declining energy expenditure (through weight loss) in tandem with increased desire in calorie consumption drive weight maintenance (instead of continued weight decline) after initial weight loss, leading to a weight plateau^1^.

As a future area of study, we propose introducing phases of caloric maintenance after a period of caloric deficit before reintroducing a caloric deficit again as a potential mechanism to combat diet fatigue. As a person transitions from a weight loss to weight maintenance phase, a shift in the approach used may be required. Strategies that were most effective for weight loss may not be the same strategies that guide successful weight maintenance^2^. A shift in motivational influences from the weight loss phase to the weight maintenance phase may also occur^34^. As a result, cognitive and emotional aspects or perceived structural support systems may largely influence weight-management success^1,34,35^. Learning weight maintenance skills and strategies (e.g., before or after weight loss) may be important for maintaining weight loss. Data from a randomized controlled trial of overweight and obese women found that learning weight maintenance “stability skills” before losing weight was more successful in helping women to maintain weight loss compared with women who lost weight first and later learned the weight maintenance approaches^36^. Employing effective, evidence-based approaches may make it easier for individuals to maintain their weight loss over time. Data from the National Weight Control Registry provides evidence that after individuals have successfully maintained their weight loss for 2–5 years, the chance of longer-term success greatly increases^2^.

Several aspects of the design were strengths of our study. First, the crossover design allowed participants to serve as their own controls. While a crossover design carries inherent problems and would typically not be used in a weight-loss randomized controlled trial, the design did allow for the novel examination of whether switching weight-loss diets instigated renewed weight loss. The lack of a washout period would usually be considered a limitation; however, in this exploratory study, the point was to examine the initial impact of switching from one diet to another, and therefore a washout would not have been warranted. Free-living energy intake is known to fluctuate widely from day to day, making long-term observations among free-living individuals important for generalizability. Studies like ours involving free-living participants are generally limited because participants are notorious for being unable to provide accurate estimates of energy intake using self-report measures. However, to overcome this limitation, this study extensively reported the design, definitions of study diets, and participant’s dietary adherence using dietary intake data obtained from 24-h dietary recalls. We also acknowledge that when weight loss is achieved, regular physical activity may contribute to the maintenance of that weight loss^37^. This study’s protocol asked participants to maintain healthy levels of physical activity through the duration of the study. Future studies may consider examining the role of an exercise-based intervention during a period of weight maintenance before participants engage in a new or different weight loss diet.

Regulation of body weight is complex and involves interactions among behavioral, physiological, environmental, and cognitive/psychosocial factors^27^. This study took an opportunity to examine whether switching diets would possibly help to address “diet fatigue”, and in the context here, it was not. Although switching diet did not further lead to weight loss, it seems that this approach at least maintained the weight loss without participants regaining significant weight. Other novel ways to promote longer-term dietary adherence should be examined. Clinicians are challenged by helping people achieve and then maintain weight loss. A task for researchers is to help clinicians by developing interventions and strategies that not only promote initial weight loss, but also contribute to subsequent weight maintenance. It will be important to continue improving our understanding of the mechanistic interactions between both physiological and behavioral factors that influence the transition from weight loss to weight plateaus and subsequently weight regain. It remains critical for future behavioral research to target specific behavioral domains or psychosocial and demographic factors that may promote longer-term adherence and thus promote successful weight maintenance^1,38^.

## Methods

Full details of the study design and outcomes from the parent study have been described elsewhere^26^. Here we briefly describe methods relevant to this analysis.

### Study design

The parent study was a 2 × 2 design assessing the effectiveness of a LF versus LC diet among insulin resistant (IR) and insulin sensitive (IS) adults who were overweight or obese^26^. We suggest the terms “insulin resistance” and “insulin sensitivity” here be interpreted cautiously because we used a proxy measure for this, rather than a direct measure (expanded discussion is provided elsewhere^26^). Briefly, insulin resistance status was assessed by calculating an area under the curve of insulin concentrations from four blood samples taken during an oral glucose tolerance test (OGTT) (time: 0, 30, 60, and 120 min)^26^. Individuals above the median (separately for men and women) were considered more IR and individuals below the median were considered more IS. Participants were then assigned to either their first diet assignment (LF or LC) via a random number generator in Microsoft Excel.

### Participants

Participants were recruited from the local Palo Alto, CA community primarily through media advertisements. Premenopausal women and men aged 18–50 years were invited to enroll if their BMI was 28–40 kg/m^2^, body weight was stable over the previous 2 months, and medications were stable for ≥ 3 months. Potential participants were excluded if they self-reported hypertension (except for those stable on antihypertension medications); type 1 or 2 diabetes mellitus; heart, renal, or liver disease; cancer or active neoplasms; hyperthyroidism unless treated and under control; taking any medications known to affect weight/energy expenditure or blood lipids; smoking; alcohol intake ≥ 3 drinks/day; pregnancy, lactation, no menstruation for the previous 12 months, or plans to become pregnant within the next year. All study participants provided written informed consent. Procedures for this study were followed in accordance with the ethical standards from the Declaration of Helsinki. The study was approved by the Stanford University Human Subjects Committee (Protocol ID: 23438, approved: 2013.12.17).

### Intervention

The intervention consisted of two 6-month phases. Participants ate an assigned diet (healthy LF or LC) for 6 months then switched to the opposite diet for an additional 6 months. During each phase, participants received 14 1-h nutrition education classes led by a health educator. Classes were delivered in-person once a week for the first 8 weeks, every other week for the next 8 weeks, and once a month for the last 8 weeks.

The curriculum of the nutrition education classes emphasized the Limbo-Titrate-Quality approach for defining a healthy LF and LC diet. There were three components of this approach. The first component was “Limbo,” or “How low can you go?” This involved participants in the LF group reducing their total daily fat intake to 20 g/day or less and participants in the LC group reducing their total daily carbohydrate intake to 20/g a day or less for the first 8 weeks. The second component, “Titrate,” involved participants incrementally adding back 5 g of fat or carbohydrates per day to their assigned diet for 1–4 weeks (e.g., going from 20 g of total fat to 25 g of total fat for the LF group). An important part of the second component was for participants to identify the lowest level of daily fat or carbohydrates they felt that they could maintain long term. The third component was “Quality,” which emphasized diet quality. Participants were encouraged to consume nutrient dense foods, fresh vegetables and fruits, and to prepare meals at home while avoiding heavily processed foods, foods with added sugars, refined white flour products, and foods with trans fats. In summary, the Limbo-Titrate-Quality approach was designed to motivate participants to achieve the lowest possible level of fat or carbohydrate intake, that is, an approach that was equally ambitious with maximal overall nutritional quality and a dietary pattern that could be continued for a lifetime.

There were no caloric restriction requirements for this dietary intervention. Nutrition education classes also addressed mindful eating, body acceptance, sugar addiction, getting adequate sleep, and maintaining healthy levels of physical activity. Participants were encouraged to track their dietary intake using daily food journals. Participants were also encouraged to be physically active and were provided with pedometers (Omron HJ-112 Digital Pocket Pedometer) to track their activity.

### Sociodemographics

Self-reported sociodemographic data on age, gender, race/ethnicity, marital status, education, and employment status were collected at baseline.

### Anthropometric measures

Participants’ current body weight was measured to the nearest 0.1 kg at each time point (baseline, 3, 6, 9, and 12 months) using a calibrated scale (Scaletronix). Participants’ height was measured at baseline to the nearest millimeter using a standard wall-mounted stadiometer. Average daily energy expenditure was assessed using the Stanford 7-day physical activity recall^39^.

### Dietary data

Dietary intake data was collected via 3 unannounced, 24-h dietary recalls within a 2-week time window at each time point (baseline, 3, 6, 9, and 12 months) using the Nutrition Data System for Research (NDS-R) software [Nutrition Coordinating Center (NCC), University of Minnesota, versions 4.05.33 (2011) and 4.06.34 (2012)]. Recalls were conducted on two weekdays and one weekend day, nonconsecutive whenever possible.

### Cardiovascular risk factors

Blood samples for analysis of plasma lipids (including high-density lipoprotein (HDL-C), low-density lipoprotein (LDL-C), and triglycerides), and insulin and glucose were collected after participants fasted for ≥ 10 h. Insulin was collected at baseline, 6, and 12 months. All other outcomes were collected at baseline, 3, 6, 9 and 12 months. HDL-C was measured by liquid selective detergent followed by enzymatic determination of cholesterol^40^. LDL-C was calculated according to Friedewald et al. equation^41^. Total plasma insulin in serum was measured by radioimmunoassay^42^, and blood glucose was measured using a modification of the glucose oxidase/peroxidase method (Diabetes Research Center, Washington University, St Louis, MO)^43,44^.

### Statistical analyses

Participant demographics and baseline clinical characteristics were summarized overall and by arm (diet order) as mean (standard deviation) or n (percent) for continuous and categorical variables, respectively.

For the primary analysis, we characterized percent weight change (PWC) before and after introducing the second diet in the crossover. The primary outcome is percent weight change at 3–6 months versus the percent weight change at 6–9 months. Absolute weight change is presented for visual observation. As prespecified in the statistical analysis plan, the primary analysis includes all available data. We fit a linear mixed model with fixed effects: categorical time (3, 6, 9, 12 months with 6 months as the reference since it is the end of the first diet and occurs before starting the new diet), order (e.g., study arm), insulin status (resistance vs sensitive), gender; and with a random effect to account for the correlated observations over time of each participant. We performed visual inspections for the model assumptions: normality of residuals and homogeneity of variance, using Q-Q plots and scatterplots, respectively. We provide model estimates for the difference in percent weight change at each time period (relative to the 6-month timepoint) along with 95% confidence intervals (CIs). These estimates account for the cross-over design and stratification randomization variables: insulin status and gender. Additionally, we also presented crude estimates for each time period, i.e., average percent weight change (95% CI). In a stratified analysis, we fit a similar linear mixed model by diet order (excluding study arm as a fixed effect) and present model estimates with 95% CIs. Additionally, we present mean percent weight change (95% CI) by diet order and insulin status.

For the secondary outcomes of LDL-C, HDL-C, triglycerides, fasting glucose, and fasting insulin, we fit a linear mixed model similar to the primary model and present model and crude estimates with 95% CIs, stratified by diet order. For blood lipids, a log transform was used on the outcome in the model to resolve departures from normality.

In a subgroup analysis, we fit a linear mixed model similar to the primary model, but included only those participants that were weight stable or gaining before the diet change at 6 months (i.e., those that can re-start weight loss). We considered participants to be weight stable or gaining if their percent weight change at the end of phase 1 (6 month timepoint) was greater than or equal to − 2 percent (weight loss) relative to the weight change at the 3-month timepoint. Estimates overall and by diet order are provided. Also, we presented crude estimates with 95% CI using only weight stable or gaining participants for the secondary outcomes.

Last, we characterized the percent weight change observed in this study (i.e., 12-month trial comparing low-fat to low-carbohydrate with a diet crossover at 6 months) and the percent weight change data from the DIETFITS dietary clinical trial (i.e., 12-month trial comparing low-fat to low-carbohydrate with the same diet for the entire study duration).

Data were analyzed in RStudio (Version 1.2.5042, RStudio Team, 2020, PBC, Boston, MA, USA).

### Supplementary Information

Supplementary Information.

## Data Availability

The data that support the findings of this study are available from the corresponding author, C.D.G., upon reasonable request.
