# Supplementary material for: Switching diets after 6-months does not result in renewed weight loss: a secondary analysis of a 12-month crossover randomized trial
Source: Sci Rep. 2024 Apr 29;14:9865. doi: 10.1038/s41598-024-60547-z (PMC11058757; doi:10.1038/s41598-024-60547-z)
Supplement: Supplementary file 1 — Supplementary Information. [file 41598_2024_60547_MOESM1_ESM.docx]

**Switching Diets After 6-months Does Not Result in Renewed Weight Loss: A Secondary Analysis of a 12-month Crossover Randomized Trial**

**Supplemental Tables and Figures**

Table of Contents

[Supplemental Figure 1. Participants change in weight 2](#_Toc156833952)

[Supplemental Figure 2. Participants change in weight by diet order 3](#_Toc156833953)

[Supplemental Figure 3. Participants change in weigh by diet order and insulin resistance at baseline 4](#_Toc156833954)

[Supplemental Table 1. Linear mixed model estimates (95% CI) for percent weight change since previous visit, relative to the reference period of 3-6 months 5](#_Toc156833955)

[Supplemental Table 2. Linear mixed model estimates (95% CI) for percent weight change since previous visit, relative to the reference period of 3-6 months, stratified by diet order 6](#_Toc156833956)

[Supplemental Table 3. Linear mixed model estimates (95% CI) for absolute weight loss relative to baseline 7](#_Toc156833957)

[Supplemental Table 4. Cardiovascular clinical measures at each time point by diet order, presented as mean (95% CI) 8](#_Toc156833958)

[Supplemental Table 5. Linear mixed model estimates (95% CI) for cardiovascular clinical measures, stratified by diet order 9](#_Toc156833959)

[Supplemental Table 6. Linear mixed model estimates (95% CI) for percent weight change since previous visit, in weight stable or weight gaining participants (n=27), stratified by diet order 10](#_Toc156833960)

**
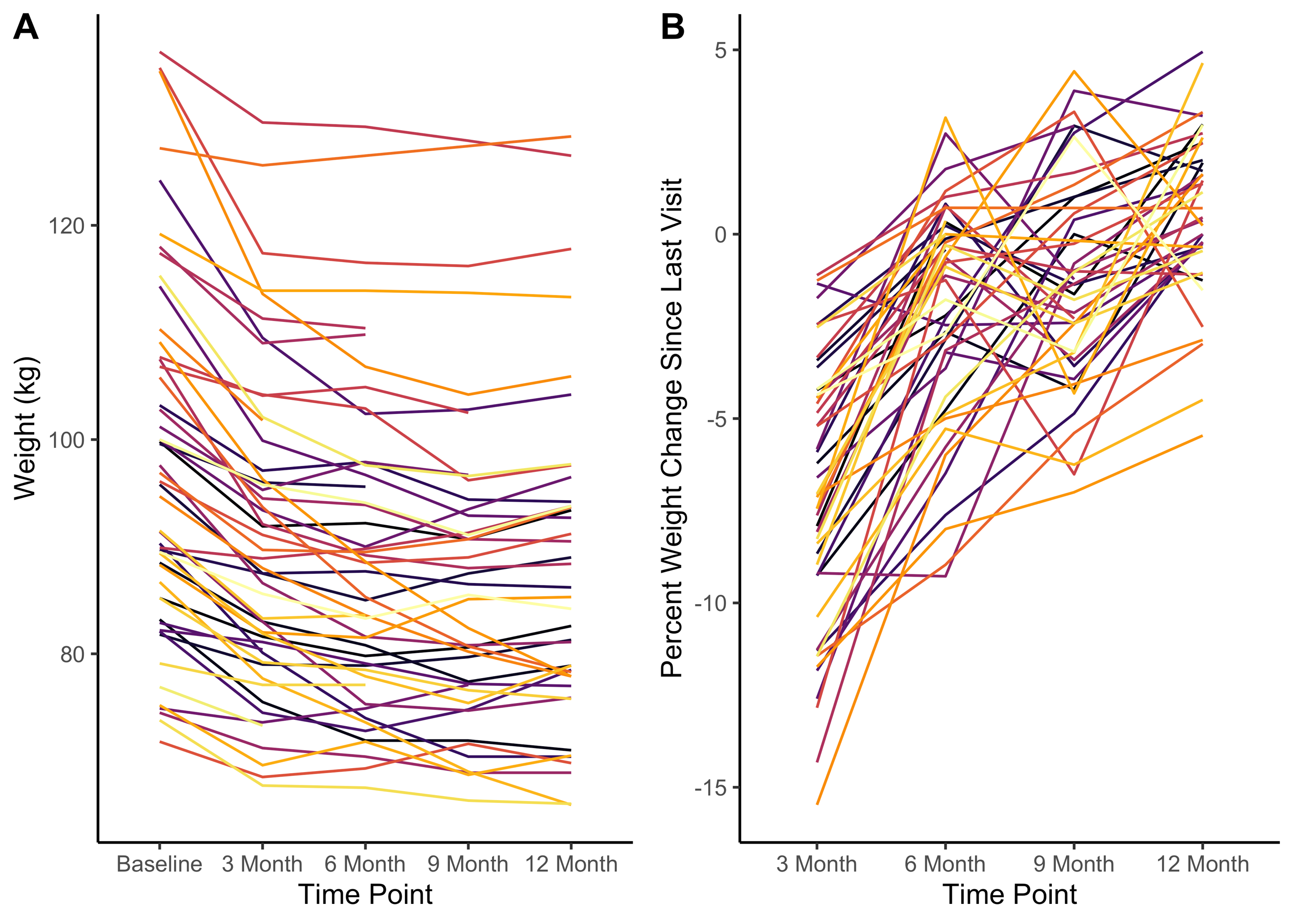
**

Supplemental Figure 1. Participants change in weight**. 1A** absolute weight (kg) and **1B** percent change in weight since last visit.


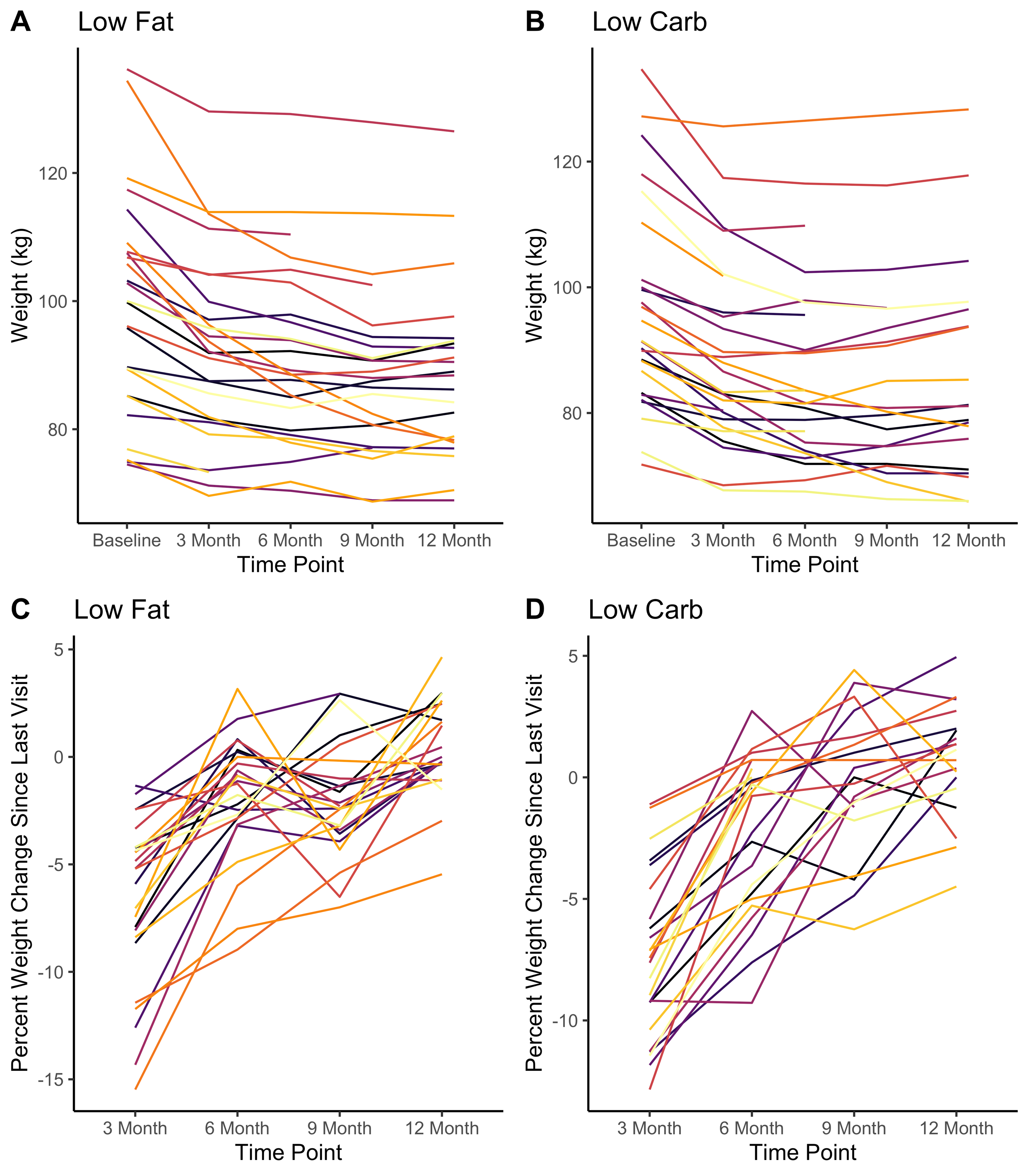


Supplemental Figure 2. Participants change in weight by diet order**. 2A and 2B** absolute weight (kg) and **2C and 2D** percent change in weight since last visit.

**
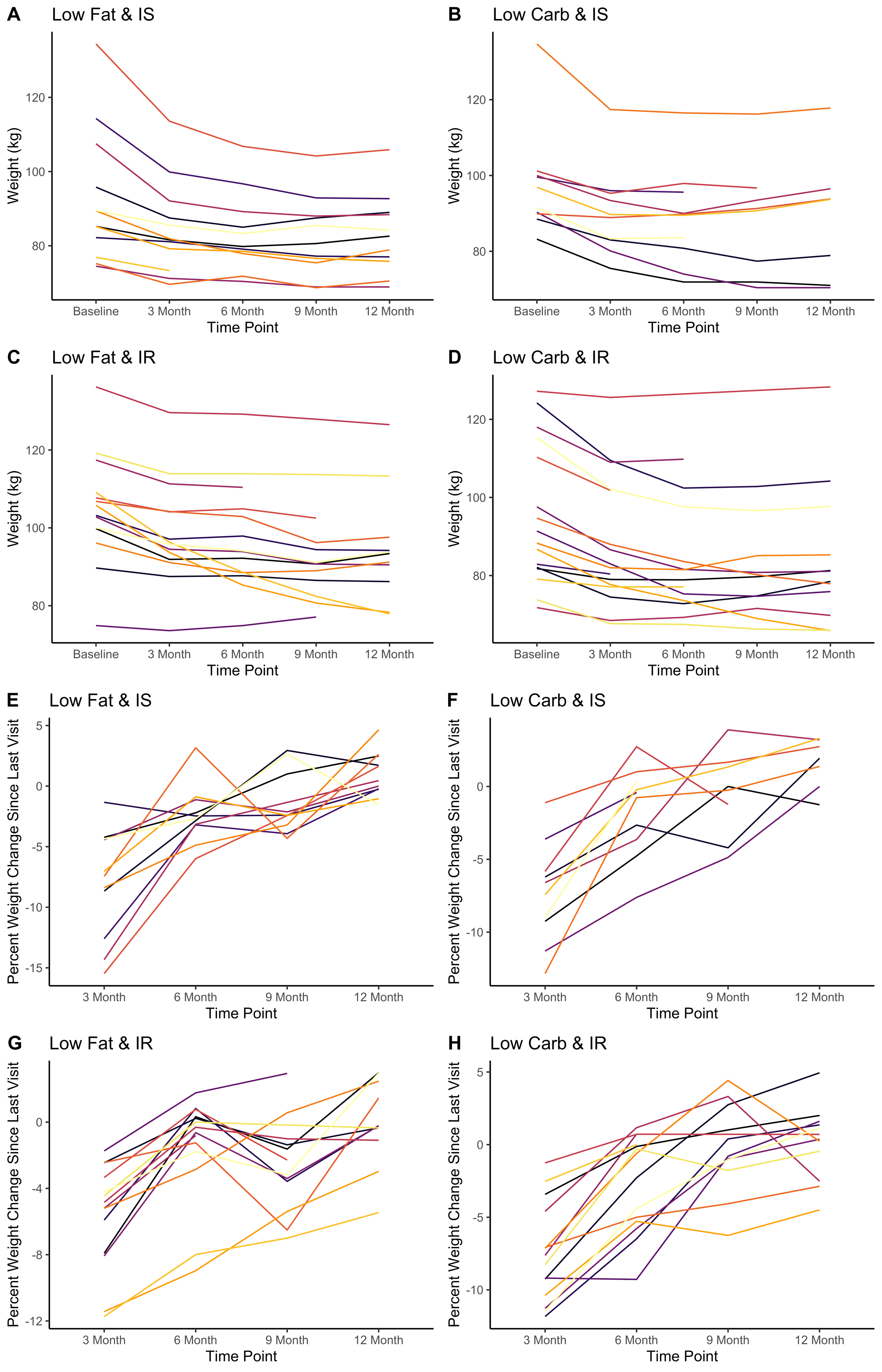
**

Supplemental Figure 3. Participants change in weigh by diet order and insulin resistance at baseline**. 3A, 3B, 3C, and 3D** absolute weight (kg) and **3E, 3F, 3G, and 3H** percent change in weight since last visit. Abbreviations: LF: Low-Fat; LC: Low-Carbohydrate; IR: Insulin Resistant; IS: Insulin Sensitive.

| Supplemental Table 1. Linear mixed model estimates (95% CI) for percent weight change since previous visit, relative to the reference period of 3-6 months | |
| --- | --- |
| **Variable** | **Model Estimate^1,2^** |
| (Intercept) | -1.6 (-2.9,-0.4) |
| Visit 3-month (first 3 months of weight change) | -4.9 (-5.9,-4.0) |
| Visit 9-month (weight change from 6-9 months) | 0.9 (-0.1,1.9) |
| Visit 12-month (weight change from 9-12 months) | 2.8 (1.8,3.9) |
| Low-Fat First | -0.3 (-1.5,1.0) |
| IS/IR status = IS | 0.0 (-1.2,1.3) |
| Gender = Male | -0.8 (-2.0,0.5) |
| ^1^ Visit at 6-months considered reference (weight change from 3-6 months)  ^2^ A negative estimate implies acceleration of weight loss whereas a positive estimate implies deceleration.  Abbreviations: IR: Insulin Resistant; IS: Insulin Sensitive | |

| Supplemental Table 2. Linear mixed model estimates (95% CI) for percent weight change since previous visit, relative to the reference period of 3-6 months, stratified by diet order | | |
| --- | --- | --- |
| **Phase 1 Diet** | **Variable** | **Model Est (95% CI)^1,2^** |
| Low-Fat | (Intercept) | -1.1(-2.6,0.4) |
|  | Visit 3 month | -4.8(-6.1,-3.4) |
|  | Visit 9 month | -0.1(-1.5,1.3) |
|  | Visit 12 month | 2.5(1.1,3.9) |
|  | IS/IR status = IS | -0.4(-2,1.3) |
|  | Gender = Male | -1.6(-3.3,0.1) |
| Low-Carbohydrate | (Intercept) | -2.3(-3.9,-0.7) |
|  | Visit 3 month | -5.1(-6.5,-3.8) |
|  | Visit 9 month | 2.2(0.7,3.6) |
|  | Visit 12 month | 3.2(1.8,4.7) |
|  | IS/IR status = IS | 0.4(-1.5,2.3) |
|  | Gender = Male | 0.1(-1.8,2) |
| ^1^ Visit at 6-months considered reference (weight change from 3-6 months)  ^2^ A negative estimate implies weight loss whereas a positive estimate implies weight gain.  Abbreviations: IR: Insulin Resistant; IS: Insulin Sensitive | | |

| Supplemental Table 3. Linear mixed model estimates (95% CI) for absolute weight loss relative to baseline | |
| --- | --- |
| **Variable** | **Model Estimate^1^** |
| (Intercept) | 91.0 (85.6,96.5) |
| Visit 3-month | -7.0 (-8.4,-5.6) |
| Visit 6-months | -8.9 (-10.3,-7.5) |
| Visit 9-month | -10.2(-11.6,-8.7) |
| Visit 12-month | -9.9 (-11.4,-8.4) |
| Low-Fat First | 5.3 (-0.5,11.1) |
| IS/IR status = IS | -6.3 (-12.1,-0.5) |
| Gender = Male | 17.9 (12,23.9) |
| ^1^ A negative estimate implies weight loss whereas a positive estimate implies weight gain.  Abbreviations: IR: Insulin Resistant; IS: Insulin Sensitive | |

| Supplemental Table 4. Cardiovascular clinical measures at each time point by diet order, presented as mean (95% CI) | | | |
| --- | --- | --- | --- |
| Clinical Measure | Time Point | Low-Fat First | Low-Carbohydrate First |
| LDL-C | Baseline | 114 (105,123) | 110 (101,120) |
|  | 3 Month | 98 (87,108) | 127 (111,143) |
|  | 6 Month | 98 (87,109) | 118 (100,136) |
|  | 9 Month | 113 (101,126) | 101 (90,112) |
|  | 12 Month | 112 (100,124) | 103 (89,118) |
| HDL-C | Baseline | 119 (108,130) | 110 (94,125) |
|  | 3 Month | 105 (95,115) | 121 (102,141) |
|  | 6 Month | 107 (96,118) | 116 (96,136) |
|  | 9 Month | 123 (109,136) | 98 (86,110) |
|  | 12 Month | 120 (105,134) | 109 (96,123) |
| Triglycerides | Baseline | 131 (110,152) | 147 (117,176) |
|  | 3 Month | 118 (98,137) | 119 (94,144) |
|  | 6 Month | 107 (87,127) | 120 (89,151) |
|  | 9 Month | 80 (67,93) | 105 (82,128) |
|  | 12 Month | 87 (74,100) | 115 (90,139) |
| Fasting Glucose | Baseline | 104 (99,109) | 100 (95,105) |
|  | 3 Month | 97 (93,100) | 99 (93,105) |
|  | 6 Month | 98 (94,101) | 97 (94,100) |
|  | 9 Month | 100 (96,105) | 96 (94,99) |
|  | 12 Month | 100 (95,106) | 98 (93,104) |
| Fasting Insulin^1^ | Baseline | 18 (16,20) | 20 (16,24) |
|  | 6 Month | 12 (9,14) | 12 (10,14) |
|  | 12 Month | 12 (9,14) | 13 (11,15) |
| ^1^ Data only available at baseline, 6-months, and 12-months  Abbreviations: LDL-C: low-density lipoprotein; HDL-C: high-density lipoprotein | | | |

| Supplemental Table 5. Linear mixed model estimates (95% CI) for cardiovascular clinical measures, stratified by diet order | | | |
| --- | --- | --- | --- |
|  |  | **Low-Fat First** | **Low-Carbohydrate First** |
| **Clinical Measure^1^** | **Variable** | **Model Est (95% CI)^2,3^** | **Model Est (95% CI)^2,3^** |
| LDL-C | (Intercept) | 99.7 (84.7,117.4) | 107.3 (92.1,125.1) |
|  | Baseline | 1.2 (1.1,1.3) | 0.9 (0.9,1) |
|  | Visit 3 month | 1.0 (0.9,1.1) | 1.1 (1,1.2) |
|  | Visit 9 month | 1.2 (1.1,1.3) | 0.9 (0.8,1) |
|  | Visit 12 month | 1.2 (1.1,1.3) | 0.9 (0.8,1) |
|  | IS/IR status = IS | 0.9 (0.7,1.1) | 1.0 (0.9,1.2) |
|  | Gender = Male | 1 (0.8,1.2) | 1.1 (0.9,1.4) |
| HDL-C | (Intercept) | 48.1 (41.2,56.3) | 55.2 (50.2,60.6) |
|  | Baseline | 1.0 (0.9,1) | 0.9 (0.9,1) |
|  | Visit 3 month | 0.9 (0.8,0.9) | 0.9 (0.9,1) |
|  | Visit 9 month | 1.2 (1.2,1.3) | 1.0 (1,1.1) |
|  | Visit 12 month | 1.2 (1.2,1.3) | 1.0 (0.9,1.1) |
|  | IS/IR status = IS | 1.1 (0.9,1.4) | 1.1 (1,1.2) |
|  | Gender = Male | 0.8 (0.7,1) | 0.7 (0.6,0.7) |
| Triglycerides | (Intercept) | 102.4 (80.9,129.6) | 94.2 (75.5,117.4) |
|  | Baseline | 1.2 (1.1,1.4) | 1.3 (1.1,1.5) |
|  | Visit 3 month | 1.1 (1,1.3) | 1.0 (0.9,1.2) |
|  | Visit 9 month | 0.8 (0.7,0.9) | 1.0 (0.9,1.2) |
|  | Visit 12 month | 0.9 (0.8,1) | 1.1 (0.9,1.3) |
|  | IS/IR status = IS | 0.8 (0.6,1) | 0.9 (0.7,1.1) |
|  | Gender = Male | 1.2 (0.9,1.5) | 1.5 (1.1,2) |
| Fasting Glucose | (Intercept) | 99.54 (93.58,105.88) | 99.71 (94.35,105.36) |
|  | Baseline | 1.07(1.02,1.12) | 1.03 (0.97,1.09) |
|  | Visit 3 month | 0.99 (0.94,1.04) | 1.02 (0.96,1.08) |
|  | Visit 9 month | 1.03 (0.98,1.08) | 1.0 (0.94,1.06) |
|  | Visit 12 month | 1.03 (0.98,1.08) | 1.01 (0.96,1.08) |
|  | IS/IR status = IS | 0.99 (0.94,1.05) | 0.99 (0.94,1.03) |
|  | Gender = Male | 0.98 (0.92,1.03) | 0.96 (0.91,1) |
| Fasting Insulin^4^ | (Intercept) | 11.6 (8.8,15.2) | 14.2 (11.2,18) |
|  | Baseline | 1.6 (1.4,1.9) | 1.6 (1.3,1.9) |
|  | Visit 12 month | 1.0 (0.8,1.2) | 1.1 (0.9,1.3) |
|  | IS/IR status = IS | 0.7 (0.5,0.9) | 0.7 (0.6,0.9) |
|  | Gender = Male | 1.2 (0.9,1.5) | 0.9 (0.7,1.1) |
| ^1^ Measures were log-transformed; for log-fasting glucose mode values deviated from normal distribution despite log transformation.  ^2^ Visit at 6-months considered reference  ^3^  A negative estimate implies weight loss whereas a positive estimate implies weight gain.  ^4^ Data only available at baseline, 6-months, and 12-months  Abbreviations: LDL-C: low-density lipoprotein; HDL-C: high-density lipoprotein; IR: Insulin Resistant; IS: Insulin Sensitive | | | |

| Supplemental Table 6. Linear mixed model estimates (95% CI) for percent weight change since previous visit, in weight stable or weight gaining participants (n=27), stratified by diet order | | | |
| --- | --- | --- | --- |
|  | **Model Estimates^1^** | | |
| **Variable** | **All Participants** | **Low-Fat First** | **Low-Carbohydrate First** |
| (Intercept) | 0.8(-0.3,1.9) | 0.0 (-1.1,1.1) | 0.3 (-1.3,2) |
| Visit 3-month | -5.5(-6.6,-4.4) | -4.6 (-5.8,-3.3) | -5.9 (-7.6,-4.1) |
| Visit 9-month | -1.0(-2.3,0.2) | -1.9 (-3.1,-0.6) | 1.2 (-0.8,3.2) |
| Visit 12-month | 0.6(-0.6,1.9) | 0.8 (-0.5,2.2) | 0.8 (-1.2,2.8) |
| Low-Fat First | -0.8(-1.8,0.2) | N/A | N/A |
| IS/IR status = IS | -0.4(-1.4,0.6) | -1.0 (-2.8,0.8) | -0.2 (-2.0,1.7) |
| Gender = Male | -0.3(-1.4,0.8) | -0.4 (-2.0,1.2) | -0.4 (-2.2,1.5) |
| ^1^ Visit at 6-months considered reference (weight change from 3-6 months)  Abbreviations: IR: Insulin Resistant; IS: Insulin Sensitive | | | |
